# Immune Biomarkers Predictive of Respiratory Viral Infection in Elderly Nursing Home Residents

**DOI:** 10.1371/journal.pone.0108481

**Published:** 2014-10-02

**Authors:** Jennie Johnstone, Robin Parsons, Fernando Botelho, Jamie Millar, Shelly McNeil, Tamas Fulop, Janet McElhaney, Melissa K. Andrew, Stephen D. Walter, P. J. Devereaux, Mehrnoush Malekesmaeili, Ryan R. Brinkman, James Mahony, Jonathan Bramson, Mark Loeb

**Affiliations:** 1 Department of Clinical Epidemiology and Biostatistics, McMaster University, Hamilton, Ontario, Canada; 2 McMaster Immunology Research Centre, McMaster University, Hamilton, Ontario, Canada; 3 Canadian Center for Vaccinology, IWK Health Centre and Capital Health, Dalhousie University, Halifax, Nova Scotia, Canada; 4 Department of Medicine, Geriatrics Division, Research Center on Aging, University of Sherbrooke, Sherbrooke, Quebec, Canada; 5 Department of Medicine, Northern Ontario School of Medicine, Sudbury, Ontario, Canada; 6 Department of Medicine, Dalhousie University, Halifax, Nova Scotia, Canada; 7 Department of Medicine, McMaster University, Hamilton, Ontario, Canada; 8 Terry Fox Laboratory, British Columbia Cancer Agency, Vancouver, British Columbia, Canada; 9 Department of Medical Genetics, University of British Columbia, Vancouver, British Columbia, Canada; 10 Department of Pathology and Molecular Medicine, McMaster University, Hamilton, Ontario, Canada; 11 Institute for Infectious Disease Research, McMaster University, Hamilton, Ontario, Canada; INSERM, France

## Abstract

**Objective:**

To determine if immune phenotypes associated with immunosenescence predict risk of respiratory viral infection in elderly nursing home residents.

**Methods:**

Residents ≥65 years from 32 nursing homes in 4 Canadian cities were enrolled in Fall 2009, 2010 and 2011, and followed for one influenza season. Following influenza vaccination, peripheral blood mononuclear cells (PBMCs) were obtained and analysed by flow cytometry for T-regs, CD4+ and CD8+ T-cell subsets (CCR7+CD45RA+, CCR7-CD45RA+ and CD28-CD57+) and CMV-reactive CD4+ and CD8+ T-cells. Nasopharyngeal swabs were obtained and tested for viruses in symptomatic residents. A Cox proportional hazards model adjusted for age, sex and frailty, determined the relationship between immune phenotypes and time to viral infection.

**Results:**

1072 residents were enrolled; median age 86 years and 72% female. 269 swabs were obtained, 87 were positive for virus: influenza (24%), RSV (14%), coronavirus (32%), rhinovirus (17%), human metapneumovirus (9%) and parainfluenza (5%). In multivariable analysis, high T-reg% (HR 0.41, 95% CI 0.20–0.81) and high CMV-reactive CD4+ T-cell% (HR 1.69, 95% CI 1.03–2.78) were predictive of respiratory viral infection.

**Conclusions:**

In elderly nursing home residents, high CMV-reactive CD4+ T-cells were associated with an increased risk and high T-regs were associated with a reduced risk of respiratory viral infection.

## Introduction

The burden of respiratory viral infection in elderly nursing home residents is high [1]. With active surveillance the incidence of respiratory viral infection is estimated to range from 1.4–2.8 per 1000 resident days [2]. Influenza and respiratory syncytial virus (RSV) are the viruses commonly responsible for morbidity and mortality associated with infection, but other respiratory viruses including parainfluenza, human metapneumovirus, coronavirus and rhinovirus can also cause severe disease in this population [1], [3]–[7]. It is a widely held belief that immunosenescence, the waning of immune function associated with old age, is responsible for this increased risk and severity of infection [8]; however, only sparse data exist to substantiate this position [9].

As a first step towards the identification of immune biomarkers predictive of respiratory viral infection in elderly nursing home residents, we characterized immune phenotypes in elderly nursing home residents [10]. Whole blood analysis of circulating CD4+ and CD8+ T-cell subsets was performed in a cross-sectional study involving 262 nursing home elderly participants and immune phenotypes were compared to immune phenotypes from healthy adults. In addition, we explored how individual immune phenotypes were influenced by age, sex, frailty and nutritional status in the nursing home elderly [10]. We observed lower naïve CD8+ T-cells (CD8+CD45RA+CCR7+) and higher terminally differentiated memory T-cells (CD8+CD45RA+CCR7-) and senescent T-cells (CD8+CD28−) when compared to healthy adults [10], consistent with prior findings in elderly people [11]–[14]. It is hypothesized that the reduced numbers of naïve CD8+ T-cells observed in the elderly due to thymic involution, coupled with an accumulation of poorly functioning terminally differentiated memory T-cells and senescent cells possibly arising from chronic antigenic stimulation by cytomegalovirus (CMV) [15], [16], predisposes elderly people to infection [17].

Supporting this hypothesis, senescent CD8+ T-cells and high titres of CMV antibody have been found to be associated with influenza vaccine failure in older people [18]–[20]. Whether these same CD4+ T-cell subsets are associated with infection is less clear. The accumulation of a separate class of T-cell, the regulatory CD4+ T-cell (T-regs) in elderly people has also been observed in elderly nursing home residents [10] and community dwelling elderly people [21]. While T-regs are known to be responsible for controlling the magnitude of CD4+ and CD8+ T-cell responses to viral infections [22], whether the accumulated T-regs in the elderly lead to impairment of host control of infection is not known.

To our knowledge, the relationship between immune phenotypes associated with immunosenescence and risk of respiratory viral infection has not been studied. If a relationship is established, this could help identify elderly nursing home residents at highest risk of become ill and could provide more focused care through targeted prevention. To this end, we sought to identify immune biomarkers predictive of respiratory viral infection during the ensuing respiratory viral season, in an elderly nursing home cohort.

## Methods

### Subjects and Setting

In this prospective cohort study, elderly participants were recruited from 32 nursing homes in 4 Canadian cities (Halifax, Nova Scotia, Sherbrooke, Quebec, Hamilton, Ontario and Vancouver, British Columbia) in September and October 2009, 2010 and 2011. Residents recruited for a separate study [10] were also eligible for inclusion in this study. Residents ≥65 years of age were eligible for the study. Exclusion criteria included individuals: not planning to be vaccinated against influenza, receiving immunosuppressive medications (including cancer chemotherapy, oral corticosteroid use >21 days, methotrexate, post-transplant medications and/or anti-cytokine or B-lymphocyte depletion therapies), or expected to die within 30 days, as determined by the supervising physician. Written informed consent was obtained from all participants or their legally appointed guardian in the event they were not competent to provide consent themselves. The study protocol was approved by the Research Ethics Board at each participating institution and nursing home.

Trained research personnel abstracted baseline demographics from the participants based on an interview, examination and chart review. Frailty was rated according to the Clinical Frailty Scale, an 8-point scale ranging from 1–8 as follows: (1) very fit, (2) well, (3) well with treated comorbid illness, (4) apparently vulnerable, (5) mildly frail defined as dependence in instrumental activities of daily living (ADL), (6) moderately frail defined as required assistance with basic ADL, (7) severely frail defined as completely dependent on others for ADL and (8) very severely frail [23]. The Clinical Frailty Scale has been validated in the nursing home population [23]. Participants received the seasonal influenza vaccine, typically in October or November, by public health nurses in accordance with guideline recommendations for the given year [24]–[26]. Peripheral blood mononuclear cells (PBMCs) were drawn from participants 21 days post vaccination.

Residents were actively followed by research staff for the influenza season immediately following the PBMC draw. The influenza season was defined as spanning from the first week ≥5% of specimens submitted to the local public health laboratory for viral testing were positive for influenza and ending when <5% were positive for influenza for 2 consecutive weeks. The influenza season was chosen as the period of follow-up as the rate of respiratory viral infection is highest during the winter months [2]. Trained research personnel reviewed each participant for the presence of symptoms or signs of respiratory illness twice weekly or more often if notified of symptoms by nursing home staff. Nasopharyngeal swabs (Copan ESwab, Copan Diagnostics Inc., Murrieta, California) were obtained by the research staff when a resident had one or more of the following new symptoms or signs: fever (≥38°Celsius), worsening cough, nasal congestion, sore throat, headache, sinus problems, muscle aches, fatigue, ear ache or infection, chills, not otherwise explained by an alternative diagnosis.

### Peripheral Blood Mononuclear Cell Analysis and Flow Cytometry

Blood was obtained from participants between 0700 and 1000 hours and hand delivered to the research laboratory for immediate processing. PBMCs were isolated and frozen using a validated common standard operating procedure [27].

T-cell immune phenotypes were determined by thawing patient PBMCs as previously described [28]. Viability of the PBMCs was found to range between 87% and 98% and the average viability was 94.6%. An aliquot (0.5–16×10^6^ cells/stain) was placed in round-bottom 96-well plates with anti-CD3-Qdot605, anti-CD8-Alexa Flour 700, anti-CD4-Pacific Blue, anti-CD45RA-PE Texas Red, anti-CD28-PE, anti-CD57-FITC, anti-CCR7-PE Cy7. T-regs were identified using anti-CD3-FITC, anti-CD4-Pacific Blue, anti-CD127-PerCP-Cy5.5, anti-CD25-PE, and anti-FoxP3-AlexaFluor700. The following antibodies were purchased from BD Bioscience: anti-CD4-Pacific Blue, anti-CD28-PE, anti-CCR7-PE-Cy7 and anti-CD25-PE. The following antibodies were purchased from eBioscience: anti-CD3-FITC, anti-CD127-PerCP-Cy5.5, anti-FoxP3-AlexaFluor700. The anti-CD3-Qdot605 was purchased from Invitrogen. The anti-CD57-FITC and anti-CD45RA-PE-TexasRed antibodies were purchased from Beckman Coulter. We defined the T-cell subsets as follows: naïve (CD45RA+CCR7+), terminally differentiated (CD45RA+CCR7-) and senescent (CD28-CD57+). T-regs were defined as CD4+CD25^hi^CD127^lo^Foxp3+. CD4+ and CD8+ immune phenotypes and T-regs were expressed as a percentage of CD3+. Antibody staining was performed using a Beckman Coulter Biomek NX^p^ Laboratory Automation Workstation (Beckman Coulter, Ontario) as previously described [29], followed by analysis using an LSR II flow cytometer with a high-throughput sampler (BD Biosciences, NJ, USA). T-regs were analyzed using FlowJo 9.6 (Treestar Inc, Ashland, OR). T-cell subset analysis employed an automated gating pipeline using the flowDensity algorithm [30]. This approach uses customized threshold calculations for the different cell subsets to mimic a manual gating scheme based on expert knowledge of hierarchical gating order and 1D density information. Population identification is individually tuned to each cell population in a data driven manner. T-cell subpopulations were identified using characteristics of their density distribution such as the number of peaks, height and width of each peak, change of the slope in the distribution curve, standard deviation, and median density (Figure S1). CD45RA thresholds were estimated based on control samples, which where then applied automatically to stimulated samples. CCR7 thresholds were estimated based on CD57+ populations given the explicit instruction that CD57+ cells are CCR7- (Figure S1). A total of 17 populations identified by this approach using high performance computing resources at the Michael Smith Genome Sciences Centre in order to reduce computational time.

CMV-reactive T-cells were identified by stimulating PBMCs with a pool of overlapping peptides spanning the immunodominant pp65 protein of CMV (PepTivator pp65, Miltenyi Biotec) according to our published protocols [28]. Briefly, thawed PBMCs were cultured overnight at 37°C and stimulated with CMV peptides (2 ug/ml) for 1 hr at 37°C. A matched set of PBMCs were stimulated with DMSO as a negative control. Brefeldin A (BD Biosciences) was then added according to the manufacturer's instructions and the cells were incubated for an additional 4 hours. The cells were stained with anti-CD4-PacificBlue and anti-CD8-AlexaFluor700, permeabilized and finally stained with anti-IFN-γ-APC, anti-TNF-α-FITC and anti-CD3-QDot605. CMV-reactive T-cells were identified as CD3+ (CD4+ or CD8+) IFN-γ+ TNF-α+.

### Respiratory Virus Detection

Using 200 ul of nasopharyngeal swab material, nucleic acid was extracted by the bioMerieux easyMAG automated extractor. Specimens were tested using the xTAG Respiratory Virus Panel (RVP) assay for influenza A (subtype H1 and H3), influenza B, RSV (subtype A and B), parainfluenza (1–4), coronavirus (NL63, OC43, HKU1 and 229E), human metapneumovirus, entero-rhinovirus, and adenovirus as per the manufacturer's protocol (Luminex Molecular Diagnostics, Inc., Toronto, Ontario).

### Statistical analysis

The immune phenotype distributions as well as the age distribution were skewed, and so the distributions of these continuous variables were summarized as medians and interquartile ranges (IQR). The age and sex for those who had PBMCs obtained and those who did not were compared using Mann-Whitney U and chi-square test as appropriate. Complete case analysis of immune phenotypes was planned if there was <10% missing data for each parameter [31].

Unadjusted hazard ratios (HR) and 95% confidence intervals (CIs) using Cox proportional hazards model were first constructed to explore the relationship between immune phenotypes and time to symptomatic respiratory viral infection. In the event a resident had multiple respiratory viral infections, only the first infection was included as an outcome. If a participant died prior to a respiratory viral infection, their time was censored on the date of death. We hypothesized that the following immune phenotypes associated with immunosenescence would be associated with increased risk of infection [12]–[17], [21]: low CD4+ and CD8+ naïve T-cells and high CD4+ and CD8+ terminally differentiated and senescent T-cells as well as high CMV-reactive CD4+ and CD8+ T-cells and high T-regs. Low was defined as immune phenotypes in the first quartile of the distribution and high was defined as immune phenotypes in the fourth quartile of the distribution. *A priori*, it was decided that age, sex and frailty would be included in the final model, given their potential for confounding with the effects of primary interest in this population [32]. Immune phenotypes with a p-value <0.20 in univariable analysis were included in the final multivariable model. The final model was determined using backwards elimination. A sandwich variance estimator was used to account for the clustering effect at the level of the nursing homes [33]. The proportional hazards assumption for continuous variables was explored graphically by plotting partial residuals against time to event and tested by regressing the partial residuals against time. The proportional hazards assumption for categorical variables was examined by a log-minus-log graph to ensure the plotted lines remained parallel. The presence of multicollinearity was examined using the variance inflation factor (VIF); presence of multicollinearity was defined as VIF >5.

P-values and 95% CIs were constructed using 2-tailed tests. P-values <0.05 were considered statistically significant. Statistical analyses were performed using R, version 3.0.2 [34].

## Results

### Nursing Home Cohort

In total, 1165 residents were enrolled in the study and of these, and PBMC were obtained from 1087 (93%). Reasons for not obtaining PBMCs were either refusal of influenza vaccine or refusal of blood draw. There was no statistically significant difference in age (median, 86 (IQR 80–90) versus 85 (IQR 79–89) without PBMCs, p = 0.42) or sex (%female, 72% versus 64% without PBMCs, p = 0.12) between those that did and did not have PBMCs obtained. Fifteen participants (1%) withdrew before the end of the study, leaving 1072 as our final sample size. Seventy-three participants died before a respiratory viral infection could be identified.

### Baseline Characteristics

**Figure 1:**
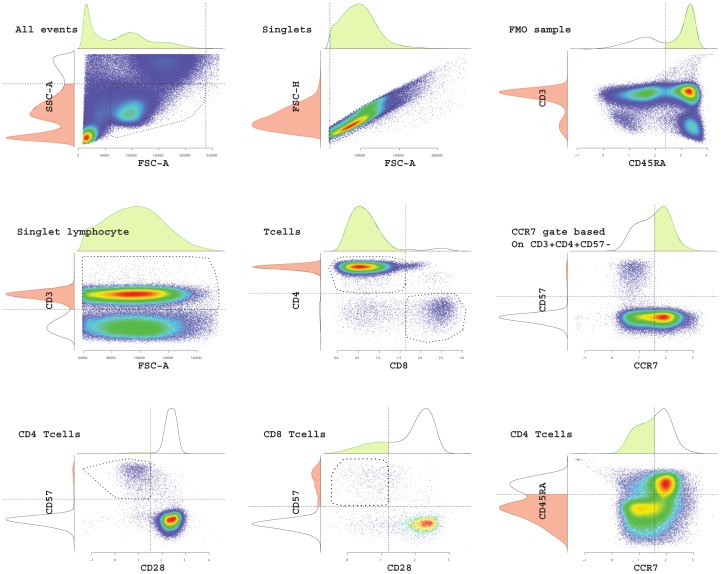
Gating strategy for T-cell phenotypes. T cell phenotypes were defined using the flowDensity software package. Lymphocytes were first gated from non-margin events, and then singlets were gated. CD45RA thresholds were calculated based on singlet lymphocytes FMO. CD3+ cells were gated and then separated into CD4+CD8- and CD4-CD8+. Expression of CD57, CD28, CD45RA and CCR7 was analyzed on either CD4+CD8- or CD8-CD4+.

**Figure 2:**
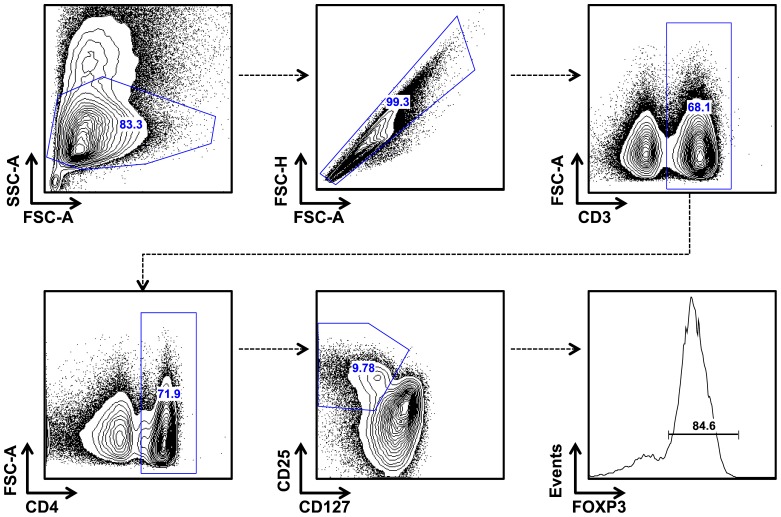
Gating strategy of T-reg. Lymphocytes and singlets were selected. Gates were then set up for CD3+ cells and CD4+. To identify the T-regs, a gate was set up to select CD25+CD127 ^lo/−^ and T-regs were defined as CD25+CD127 ^lo/−^ FOXP3+.

**Figure 3:**
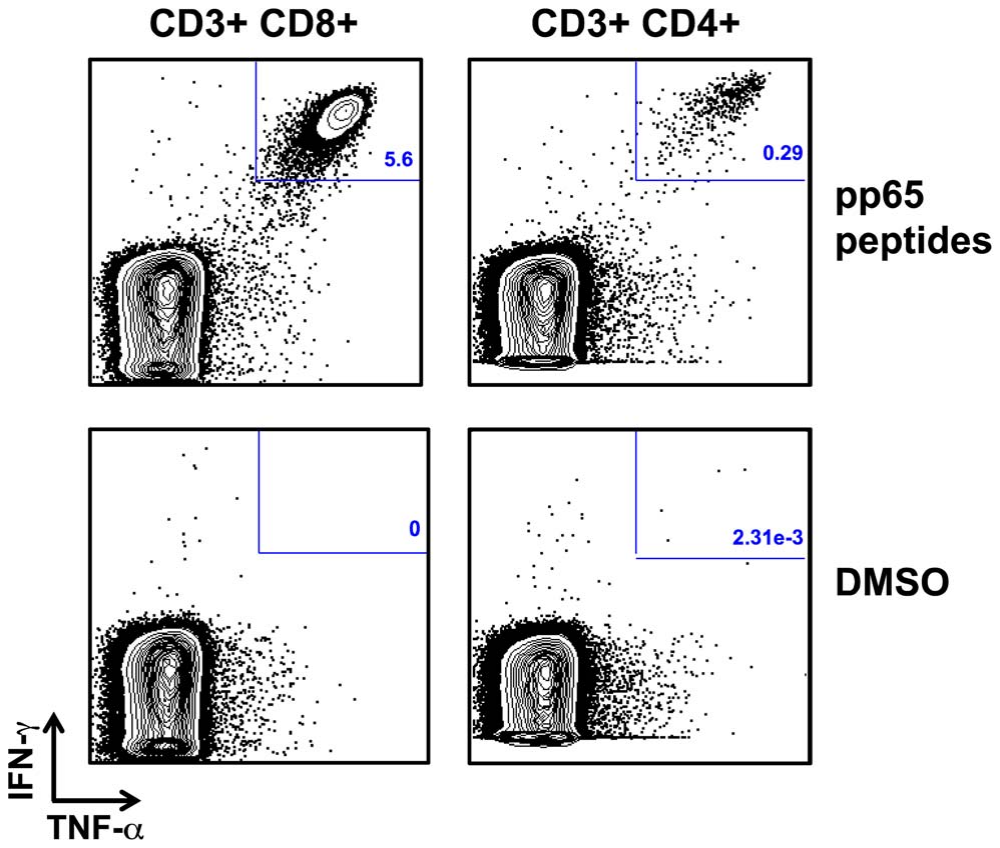
Gating strategy for CMV-reactive T cells. PBMC were stimulated with pp65 peptides to identify CMV-reactive T-cells. As a negative control, PBMC were stimulated with DMSO. Subsequently, the T-cells were stained for surface markers and intracellular cytokines. To define the CMV-reactive T-cells, the flow data was gated on singlet lymphocytes (as shown in Figures 1 and 2) and subsequently gated on CD3+CD8+ cells and CD3+CD4+. The plots show intracellular cytokine staining results for a single patient. CMV-reactive T-cells were defined as IFN-γ+ TNF-α+.

Baseline characteristics of the final cohort are summarized in Table 1. The median age was 86 years (IQR 80–90 years) and the ages ranged from 65–102 years. Eight persons were ≥100 years. Most (93%) had at least one co-morbidity, and almost half (44%) scored either 7 or 8 on the Clinical Frailty Scale, which is defined as severely and very severely frail respectively. Figures 1, 2 and 3 describe the gating strategies used to define the immune phenotypes. The medians and corresponding IQRs for each immune cell type tested in this study can be found in Table 1.

**Table 1:** Baseline characteristics of the nursing home elderly.

	Total n = 1072
**Demographics [n(%)]**	
Age (years)	
65–74	131 (12)
75–84	337 (31)
85–94	508 (47)
≥95	96 (9)
Sex (F)	776 (72)
Prior co-morbidity	
COPD	186 (17)
Coronary artery disease	346 (32)
Diabetes	290 (27)
Heart failure	148 (14)
Stroke	273 (25)
Dementia	511 (48)
≥5 medications	966 (90)
Frailty	
4	76 (7)
5	174 (16)
6	354 (33)
7	460 (43)
8	8 (1)
**Immune Phenotypes [median (IQR)]**	
CD8+ T-cell	
Naïve CD8+ T-cell%	1.10 (0.60–1.82)
Terminally differentiated CD8+ T-cell%	8.95 (4.72–14.80)
Senescent CD8+ T-cell%	5.87 (2.40–11.58)
CD8+ CMV T-cell%	0.32 (.03–1.53)
CD4+ T-cell	
Naïve CD4+ T-cell%	13.2 (6.90–22.85)
Terminally differentiated CD4+ T-cell%	8.46 (4.93–14.04)
Senescent CD4+ T-cell%	1.66 (0.28–4.54)
CD4+ CMV T-cell%	0.06 (0.006–0.40)
T-reg	
T-reg%	2.73 (2.12–3.45)

### Respiratory Virus Infection

In total, 269 swabs were obtained from 233 symptomatic people. Nasopharyngeal swabs were positive for viruses in 87 symptomatic residents (Table 2). Coronavirus (32%), influenza (24%), rhinovirus (17%) and RSV (14%) were the most common viruses found. One nasopharyngeal swab positive for influenza A also had rhinovirus present.

**Table 2:** Respiratory viruses present in symptomatic elderly nursing home residents.

	Nasopharyngeal swabs positive for respiratory virus* n = 87 n(%)
I**nfluenza**	21 (24)
Influenza A	16 (18)
Influenza B	5 (6)
**RSV**	12 (14)
RSV A	10 (11)
RSV B	2 (2)
**Coronavirus**	28 (32)
Coronavirus OC43	15 (17)
Coronavirus NL63/229E	9 (10)
Coronavirus HKU1	4 (5)
**Rhinovirus**	15 (17)
**Human metapneumovirus**	8 (9)
**Parainfluenza**	4 (5)
Parainfluenza 1	3 (3)
Parainfluenza 2	1 (1)

*One patient had mixed influenza A and rhinovirus.

### Predictors of Respiratory Virus Infection

**Figure 4:**
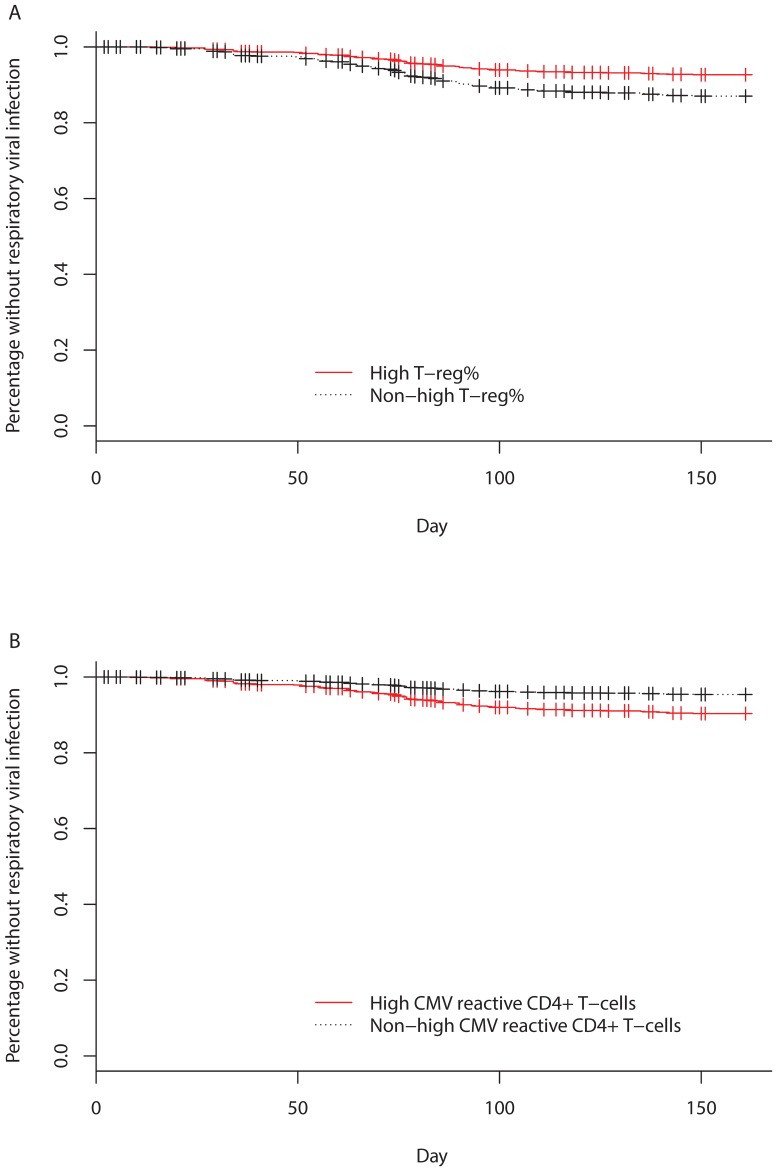
Time to respiratory viral infection stratified by a) T-reg%, adjusted for age, sex, frailty and CMV-reactive CD4+ T-cell%) and b) CMV-reactive CD4+ T-cell%, adjusted for age, sex, frailty and T-reg%.

We subsequently investigated whether a relationship existed between specific immune cell populations and respiratory virus infection. The proportional hazards assumption was satisfied for all covariates included in the model and there was no concerning evidence of multicollinearity (all VIFs were <3). In univariable analyses, low naïve CD8+ T-cell% (HR 0.69, 95% CI 0.51–0.95) and high T-reg% (HR 0.47, 95% CI 0.26–0.85) were associated with a reduced risk of respiratory viral infection and high terminally differentiated CD8+ T-cell% (HR 1.57, 95% CI 1.10–2.24), high senescent CD8+ T-cell% (HR 1.55, 95% CI 1.11–2.17) and high CMV-reactive CD4+ T-cell% (HR 1.82, 95% CI 1.13–2.94), were associated with an increased risk of respiratory viral infection (Table 3). In multivariable analysis adjusted for age, sex and frailty, only high T-reg% (HR 0.41, 95% CI 0.20–0.81) and high CMV-reactive CD4+ T-cell% (HR 1.69, 95% CI 1.03–2.78) remained predictive of respiratory viral infection in the final model (Table 3 and Figure 4).

**Table 3:** Immune phenotype predictors of respiratory viral infection in univariable and multivariable analysis.

	HR (95% CI) Unadjusted	P-value	HR (95% CI) Final Model*	P-value
**Age**	0.99 (0.97–1.01)	0.35	0.99 (0.98–1.01)	0.30
**Sex**				
Male	Reference		Reference	
Female	1.13 (0.65–1.98)	0.66	1.03 (0.58–1.84)	0.92
**Clinical Frailty Scale**				
4	Reference		Reference	
5	1.44 (0.34–6.17)	0.62	2.68 (0.58–12.47)	0.21
6	1.99 (0.59–6.70)	0.27	3.67 (1.06–12.67)	0.04
7 or 8	1.41 (0.39–5.07)	0.60	2.45 (0.55–10.86)	0.24
**CD8+ T-cell**				
Low naïve CD8+ T-cell%	0.69 (0.51–0.95)	0.02		
High terminally differentiated CD8+ T-cell%	1.57 (1.10–2.24)	0.01		
High senescent CD8+ T-cell%	1.55 (1.11–2.17)	0.01		
High CMV-reactive CD8+ T-cell%	1.15 (0.65–2.03)	0.64		
**CD4+ T-cell**				
Low naïve CD4+ T-cell%	0.85 (0.61–1.18)	0.33		
High terminally differentiated CD4+ T-cell%	0.96 (0.60–1.55)	0.88		
High senescent CD4+ T-cell%	1.08 (0.71–1.64)	0.73		
High CMV-reactive CD4+ T-cell%	1.82 (1.13–2.94)	0.01	1.69 (1.03–2.78)	0.04
**T-reg**				
High T-reg%	0.47 (0.26–0.85)	0.01	0.41 (0.20–0.81)	0.01

*Final model adjusted for age, sex, frailty, high T-reg% and high CMV-reactive CD4+ T-cell%.

## Discussion

In this prospective cohort study of elderly nursing home residents, CD4+ T cells, in particular T-regs and CMV-reactive CD4+ T-cells were predictive of respiratory viral infection during the ensuing respiratory viral season in multivariable analysis. In contrast, CD8+ T-cells were not found to be predictive in multivariable analysis. To our knowledge, this is the first study to identify immune biomarkers predictive of respiratory viral infection in elderly people.

T-regs are responsible for creating the balance between the immune response to pathogens and the harmful sequelae of inflammation that arises with this response [35]. High T-regs have been consistently observed in elderly people when compared to healthy adults [10], [21], and it has been hypothesized that this shift may be associated with increased risk of infection seen in the elderly [21], [36]. It is intriguing that in our study higher levels of circulating T-regs were associated with reduced risk of symptomatic respiratory virus infection. We did not systematically test all residents in our study for respiratory virus throughout the study, so we cannot determine whether the association between high T-regs and reduced risk of infection was due to absence of infection or whether there was a higher incidence of asymptomatic infections in the high T-reg group. Little is known about the role of T-regs in preventing acute respiratory viral infection in humans [35]. In mice, a robust T-reg response has been observed during influenza [37] and RSV infection [38] and depletion of T-regs delays RSV viral clearance from lungs [38] suggesting that T-regs play an important role in controlling the immune response to respiratory viral infection. In aged mice, higher percentages of T-regs are observed at baseline and during acute influenza infection when compared to younger mice, and their presence is thought to contribute to a decrease and delay of CD8+ T-cell response during acute influenza infection [39]. In consideration of the murine data, we speculate that elevated levels of T-regs may suppress immune pathology associated with anti-viral immunity.

CMV has been proposed as the chronic antigenic stimulus responsible for accelerated immunosenescence, including the accumulation of senescent CD8+ T-cells [40], [41]. There have been at least three studies looking at the association between CMV infection and influenza vaccine response in the elderly [20], [42], [43]. In one, CMV was associated with influenza vaccine non-response [20], however two other studies found no association [42], [43]. In contrast to the reports on seropositivity, we focused on the T-cell response to CMV and observed that elevated frequencies of CMV-reactive CD4+ T-cells, but not CMV-reactive CD8+ T-cells, were associated with an increased risk of respiratory viral infection. We are unaware of any other study linking CMV-reactive CD4+ T-cells to increased risk of respiratory viral infection. It is difficult to speculate on the possible biological relationship between the CMV-reactive CD4+ T cells and susceptibility to infection. Given the observation that CD4+ T-regs also correlate with susceptibility, we interpret these collective data as an indication that the distribution of functional cells (i.e. effector, suppressor, Th1, Th2, etc…) within the CD4+ T cell compartment has a strong influence on host resistance in the elderly. Most research to date has focused on CD8+ T-cells in the elderly and these observation strongly support a new line of research in the elderly to understand how and why skewing of the CD4+ T-cell compartment contributes significantly to the outcome of respiratory infection.

Low naïve CD8+ T-cells, high terminally differentiated CD8+ T-cells and senescent CD8+ T-cells have been described in elderly populations [10]–[14] and have been hypothesized to predict risk of infection [9], [17]. Although there were associations between these immune phenotypes and risk of respiratory viral infection in univariable analyses, after adjustment for known confounders such as age, sex and frailty, CD8+ T cells were not predictive of respiratory viral infection once all immune phenotypes with suspected association with respiratory viral infection were included in the model. This illustrates the need for a robust statistical approach, including adequate sample size allowing for adjustment for known confounders and other immune phenotypes when exploring associations between immune biomarkers and outcomes.

Frailty is a “state variable” which aims to capture a person's vulnerability to adverse health outcomes. The Clinical Frailty Scale used here has been previously validated in nursing home residents and has been shown to robustly predict outcomes including mortality, disability and cognitive decline [23]. Frailty influences health outcomes through a number of mechanisms, including overall burden of disease/comorbidity and reduced reserve to tolerate further insults. Frailty has also been associated with immunosenescence [44]. Because of its importance as a measure of overall health and its relevance to immune function, it was a relevant measure to include in this study.

Our analysis was greatly facilitated by an automated analysis approach which eliminated what would have otherwise been an extremely time-consuming process of manual gating over 1,000 FCS files using an approach that was unbiased relative to manual gating with variability as low or even lower than manual gating. This approach should help facilitate the efficiency of future large studies of immune biomarkers.

This study provides insights into the role of immunosenescence and the risk of respiratory viral infection in elderly nursing home residents. Although our study was designed to identify associations and not causation, our findings suggest the possibility that strategies to boost circulating T-regs [45] or vaccines to prevent infection with CMV [46] or prophylactic anti-viral therapy to prevent re-activation of CMV may reduce respiratory viral infections in this high-risk population. In addition, those identified at increased risk of respiratory viral infection could be offered alternative prevention strategies such as heightened surveillance during the highest risk periods, which could help prevent nursing home outbreaks and transmission to healthcare workers and their families.

Limitations of this study include lower than expected influenza viral infection. Although influenza is not necessarily the most common virus isolated in nursing homes, [1], we expected to see more than 21 cases in 1072 residents during the influenza season based on prior respiratory viral infection surveillance studies conducted in nursing homes [1], [2]. We do not believe that cases were missed. Indeed, we performed prospective active surveillance for symptomatic respiratory viral infection, and approximately one third of the nasopharyngeal swabs were positive for virus, comparable to another study, which performed active surveillance for respiratory infections in nursing homes in Canada [2]. Instead, we believe the lower numbers were due to circulation of pandemic H1N1, an influenza strain that had less impact on older people during the 2009–2010 influenza season than pre-pandemic years and the relatively low incidence of influenza during 2010-2011 influenza season [47]. In addition, the lower numbers could have been due to the fact that we only included residents who had been vaccinated against influenza. We felt that influenza vaccination status was too important a confounder to manage statistically, both because of its potential ability to prevent influenza infection and because of its association with the healthy user bias [48], [49]. It remains possible that there are different immune predictors for each of the different respiratory viruses in this vaccinated cohort and combining the respiratory viruses together in one combined endpoint limited its generalizability; however we chose *a priori* to combine the respiratory viruses together in a combined endpoint based on the fact they result in similar outcomes in this population [1], [3]–[7]. The low number of participants with influenza precluded our ability to perform a sensitivity analysis, looking at immune phenotypes predictive of influenza infection.

An additional limitation was that we were unable to include immune phenotypes as continuous variables in the analysis. In general, maintaining data as continuous is preferred over categorizing data [50]; however an analysis including immune phenotype data as continuous was not feasible as it led to estimates with wide confidence intervals. Thus, the analysis was performed using categorized variables, similar to other aging studies [51]–[55]. Although seventy-three participants died prior to developing a respiratory viral infection, we do not believe this competing risk introduced significant bias. Competing risks are unlikely to bias the result when the follow-up is short, or the proportion of participants experiencing a competing risk is less than the proportion of participants experiencing the outcome [56]. In this study, the follow-up was short (only one influenza season) and the proportion of participants who died was less than those who developed a respiratory viral infection. Last, these results were obtained in a frail elderly population and may not be generalizable to community dwelling elderly.

In conclusion, in elderly nursing home residents, high CMV-reactive CD4+ T-cells were predictive of increased risk of respiratory viral infection and high T-regs were predictive of reduced risk during the ensuing respiratory viral season. These findings provide insights into immunosenescence and risk of infection and may help guide future prevention strategies.

## Supporting Information

Figure S1 **Comparison of automated versus manual gating for T-cell phenotypes.** The same gating hierarchy was used for manual (top row) and automated (bottom row) approaches for the T-cell panel as described in the text. Similar results were obtained for both methods.
(TIFF)

## References

[1] Falsey A, Dallal G, Formica M, Andolina G, Hamer D, et al (2008) Long-term care facilities: a cornucopia of viral pathogens. J Am Geriatr Soc 56: 1281–1285. 18557966 10.1111/j.1532-5415.2008.01775.x PMC2875942

[2] Loeb M, McGeer A, McArthur M, Peeling RW, Petric M, et al (2000) Surveillance for outbreaks of respiratory tract infections in nursing homes. CMAJ 162: 1133–1137. 10789627 PMC1232363

[3] Drinka P, Gravenstein S, Langer E, Krause P, Shult P (1999) Mortality following isolation of various respiratory viruses in nursing home residents. Infect Control Hosp Epidemiol 20: 812–815. 10614604 10.1086/501589

[4] Louie J, Yagi S, Nelson F, Kiang D, Glaser C, et al (2005) Rhinovirus outbreak in a long-term care facility for elderly persons associated with unusually high mortality. Clin Infect Dis 41: 262–265. 15983926 10.1086/430915 PMC7107978

[5] Hicks L, Shepard C, Britz P, Erdman D, Fischer M, et al (2006) Two outbreaks of severe respiratory disease in nursing homes associated with rhinovirus. J Am Geriatr Soc 54: 284–289. 16460380 10.1111/j.1532-5415.2005.00529.x

[6] Falsey A, McCann R, Hall W, Criddle M, Formica M, et al (1997) The “common cold” in frail older person: impact of rhinovirus and coronavirus in a senior daycare center. J Am Geriatr Soc 45: 706–711. 9180664 10.1111/j.1532-5415.1997.tb01474.x PMC7166646

[7] Boivin G, De Serres G, Hamelin M, Côté S, Argouin M, et al (2007) An outbreak of severe respiratory tract infection due to human metapneumovirus in a long-term care facility. Clin Infect Dis 44: 1152–1158. 17407031 10.1086/513204

[8] Smith P, Bennett G, Bradley S, Drinka P, Lautenbach E, et al (2008) SHEA/APIC Guideline: Infection prevention and control in the long-term care facility; Society for Healthcare Epidemiology of America (SHEA); Association for Professionals in Infection Control and Epidemiology (APIC). Am J Infect Control 36: 504–535. 18786461 10.1016/j.ajic.2008.06.001 PMC3375028

[9] Fulop T, Pawelec G, Castle S, Loeb M (2009) Immunosenescence and vaccination in nursing home residents. Clin Infect Dis 48: 443–448. 19140756 10.1086/596475

[10] Johnstone J, Millar J, Lelic A, Verschoor C, Walter S, et al (2014) Immunosenescence in the nursing home elderly. BMC Geriatrics 14: 50. 24742120 10.1186/1471-2318-14-50 PMC4013821

[11] Saule P, Trauet J, Dutriez V, Lekeux V, Dessaint J, et al (2006) Accumulation of memory T cells from childhood to old age: central and effector memory cells in CD4+ versus effector memory and terminally differentiated memory cells in CD8+ compartment. Mech Ageing Dev 127: 274–281. 16352331 10.1016/j.mad.2005.11.001

[12] Fahey J, Schnelle J, Boscardin J, Thomas J, Gorre M, et al (2000) Distinct categories of immunologic changes in frail elderly. Mech Ageing Dev 115: 1–20. 10854626 10.1016/s0047-6374(00)00094-4

[13] Effros R, Boucher N, Porter V, Zhu X, Spaulding C, et al (1994) Decline in CD28+ T cells in centenarians and in long-term T cell cultures: a possible cause for both in vivo and in vitro immunosenecence. Exp Gerontol 29: 601–609. 9435913 10.1016/0531-5565(94)90073-6

[14] Boucher N, Dufeu-Duchesne T, Vicaut E, Farge D, Effros R, et al (1998) CD28 expression in T cell aging and human longevity. Exp Gerontol 33: 267–282. 9615924 10.1016/s0531-5565(97)00132-0

[15] Wikby A, Johansson B, Olsson J, Löfgren S, Nilsson B, et al (2002) Expansion of peripheral blood CD8 T-lymphocyte subpopulations and an association with cytomegalovirus seropositivity in the elderly: the Swedish NONA immune study. Exp Gerontol 37: 445–453. 11772532 10.1016/s0531-5565(01)00212-1

[16] Looney R, Falsey A, Campbell D, Torres A, Kolassa J, et al (1999) Role of cytomegalovirus in the T-cell changes seen in elderly individuals. Clin Immunol 90: 213–219. 10080833 10.1006/clim.1998.4638

[17] Fulop T, Larbi A, Wikby A, Mocchegiani E, Hirokawa K, et al (2005) Dysregulation of T-cell function in the elderly: scientific basis and clinical implications. Drugs Aging 22: 589–603. 16038574 10.2165/00002512-200522070-00005

[18] Goronzy J, Fulbright J, Crowson C, Poland G, O'Fallon W, et al (2001) Value of immunological markers in predicting responsiveness to influenza vaccination in elderly individuals. J Virol 75: 12182–12187. 11711609 10.1128/JVI.75.24.12182-12187.2001 PMC116115

[19] Saurwein-Teissl M, Lung T, Marx F, Gschösser C, Asch E, et al (2002) Lack of antibody production following immunization in old age: association with CD8+CD28- T cell clonal expansions and an imbalance in the production of Th1 and Th2 cytokines. J Immunol 168: 5893–5899. 12023394 10.4049/jimmunol.168.11.5893

[20] Trzonkowski P, Mysliwska J, Szmit E, Wieckiewicz J, Lukaszuk K, et al (2003) Association between cytomegalovirus infection, enhanced proinflammatory response and low level of anti-hemagglutinins during the anti-influenza vaccination – an impact of immunosenescence. Vaccine 21: 3826–3836. 12922116 10.1016/s0264-410x(03)00309-8

[21] Wang L, Xie Y, Zhu L, Chang T, Mao Y, et al (2010) An association between immunosenescence and CD4+CD25+ regulatory T-cells: a systematic review. Biomed Environ Sci 23: 327–332. 20934123 10.1016/S0895-3988(10)60072-4

[22] Rowe J, Ertelt J, Way S (2012) Foxp3+ regulatory T-cells, immune stimulation and host defence against infection. Immunology 136: 1–10. 22211994 10.1111/j.1365-2567.2011.03551.x PMC3372751

[23] Rockwood K, Abeysundera M, Mitnitski A (2007) How should we grade frailty in nursing home patients? J Am Med Dir Assoc 8: 595–603. 17998116 10.1016/j.jamda.2007.07.012

[24] Langley J, Warshawsky B, Ismail S, Crowcroft N, Hanrahan A, et al (2009) Statement on seasonal trivalent inactivated influenza vaccine for 2009–2010. CCDR 35: ACS–6.

[25] Langley J, Warshawsky B, Ismail S, Crowcroft N, Hanrahan A, et al (2010) Statement on seasonal trivalent inactivated influenza vaccine for 2010–2011. CCDR 36: ACS–6. 10.14745/ccdr.v36i00a06 PMC6802438 31682656

[26] Langley J, Warshawsky B, Cooper C, Crowcroft N, Hanrahan A, et al (2011) Statement on seasonal trivalent inactivated influenza vaccine for 2011–2012. CCDR 37: ACS–5.

[27] Disis M, dela Rosa C, Goodell V, Kuan L, Chang J, et al (2006) Maximizing the retention of antigen specific lymphocyte function after cryopreservation. J Immunol 308: 13–18. 10.1016/j.jim.2005.09.011 16337957

[28] Lelic A, Verschoor C, Ventresca M, Parsons R, Evelegh C, et al (2012) The polyfunctionality of human memory CD8+ T cells elicited by acute and chronic virus infections is not influenced by age. PLoS Pathog 8: e1003076. 23271970 10.1371/journal.ppat.1003076 PMC3521721

[29] Verschoor C, Johnstone J, Millar J, Dorrington M, Habibagahi M, et al (2013) Blood CD33(+)HLA-DR(-) myeloid-derived suppressor cells are increased with age and a history of cancer. J Leukoc Biol 93: 633–637. 23341539 10.1189/jlb.0912461 PMC3701116

[30] Submitted as an R package in Bioconductor: An open source, open development software project to provide tools for the analysis and comprehension of high-throughput genomic data.

[31] Marshall A, Altman D, Holder R (2010) Comparison of imputation methods for handling missing covariate data when fitting a Cox proportional hazards model: a resampling study. BMC Med Res Methodol 31: 112. 10.1186/1471-2288-10-112 PMC3019210 21194416

[32] Fulop T, Larbi A, Witkowski J, McElhaney J, Loeb M, et al (2010) Aging, frailty and age-related disease. Biogerontology 11: 547–563. 20559726 10.1007/s10522-010-9287-2

[33] Wei L, Lin D, Weissfeld L (1989) Regression-analysis of multivariate incomplete failure time data by modeling marginal distributions. J Am Stat Assoc 84: 1065–1073.

[34] R Core Team (2013) R: A language and environment for statistical computing. R Foundation for Statistical Computing, Vienna, Austria. ISBN 3-900051-07-0. Available: http://www.R-project.org/.

[35] Keynan Y, Card C, McLaren P, Dawood M, Kasper K, et al (2008) The role of regulatory T-cells in chronic and acute viral infections. Clin Infect Dis 46: 1046–1052. 18444822 10.1086/529379

[36] Lages C, Suffia I, Velilla P, Huang B, Warshaw G, et al (2008) Functional regulatory T cells accumulate in aged hosts and promote chronic infectious disease reactivation. J Immunol 181: 1835–1848. 18641321 10.4049/jimmunol.181.3.1835 PMC2587319

[37] Betts R, Prabhu N, Ho A, Lew F, Hutchinson P, et al (2012) Influenza A virus infection results in a robust, antigen-responsive, and widely disseminated Fox3+ regulatory T cell response. J Virol 86: 2817–2825. 22205730 10.1128/JVI.05685-11 PMC3302292

[38] Fulton R, Meyerholz D, Varga S (2010) Foxp3+ CD4 regulatory T cells limit pulmonary immunopathology by modulating the CD8 T cell response during respiratory syncytial virus infection. J Immunol 185: 2382–2392. 20639494 10.4049/jimmunol.1000423 PMC2923480

[39] Williams-Bey Y, Jiang J, Murasko D (2011) Expansion of regulatory T cells in aged mice following influenza infection. Mech Ageing Dev 132: 163–170. 21414341 10.1016/j.mad.2011.03.001 PMC3111029

[40] Koch S, Larbi A, Ozcelik D, Solana R, Gouttefangeas C, et al (2007) Cytomegalovirus: A driving force in human T-cell immunosenescence. Ann NY Acad Sci 1114: 23–25. 17986574 10.1196/annals.1396.043

[41] Pawelec G, Derhovanessian E, Larbi A, Strindhall J, Wikby A, et al (2009) Cytomegalovirus and human immunosenescence. Rev Med Virol 19: 47–56. 19035529 10.1002/rmv.598

[42] den Elzen W, Vossen A, Cools H, Westendorp R, Kroes A, et al (2011) Cytomegalovirus infection and responsiveness to influenza vaccination in elderly residents of long-term care facilities. Vaccine 29: 4869–4874. 21497631 10.1016/j.vaccine.2011.03.086

[43] Derhovanessian E, Theeten H, Hahnel K, Van Damme P, Cools N, et al (2013) Cytomegalovirus-associated accumulation of late-differentiated CD4 T-cells correlates with poor humoral response to influenza vaccination. Vaccine 31: 685–690. 23196209 10.1016/j.vaccine.2012.11.041

[44] McElhaney J, Zhou X, Talbot H, Soethout E, Bleackley R, et al (2012) The unmet need in the elderly: How immunosenescence, CMV infection, co-morbidities and frailty are a challenge for the development of more effective influenza vaccines. Vaccine 30: 2060–2067. 22289511 10.1016/j.vaccine.2012.01.015 PMC3345132

[45] Sehrawat S, Rouse B (2011) Tregs and infections: on the potential value of modifying their function. J Leukoc Biol 90: 1079–1087. 21914856 10.1189/jlb.0611271 PMC3236550

[46] Krause P, Bialek S, Boppana S, Griffiths PD, Laughlin C, et al (2013) Priorities for CMV vaccine development. Vaccine 32: 4–10. 24129123 10.1016/j.vaccine.2013.09.042 PMC4623576

[47] Mitchell R, Taylor G, McGeer A, Frenette C, Suh K, et al (2013) Understanding the burden of influenza infection among adults in Canadian hospitals: a comparison of the 2009–2010 pandemic season with the prepandemic and postpandemic seasons. Am J Infect Control 41: 1032–1037. 24176768 10.1016/j.ajic.2013.06.008

[48] Jackson L, Jackson M, Nelson J, Neuzil K, Weiss N (2006) Evidence of bias in estimates of influenza vaccine effectiveness in seniors. Int J Epidemiol 35: 337–344. 16368725 10.1093/ije/dyi274

[49] Jackson L, Nelson J, Benson P, Neuzil K, Reid R, et al (2006) Functional status is a confounder of the association of influenza vaccine and risk of all cause mortality in seniors. Int J Epidemiol 35: 345–352. 16368724 10.1093/ije/dyi275

[50] Bennette C, Vickers A (2012) Against quantiles: categorization of continuous variables in epidemiologic research, and its discontents. BMC Med Res Methodol 12: 21. 22375553 10.1186/1471-2288-12-21 PMC3353173

[51] Izaks G, Remarque E, Becker S, Westendorp R (2003) Lymphocyte count and mortality risk in older persons. The Leiden 85-plus study. J Am Geriatr Soc 51: 1461–1465. 14511169 10.1046/j.1532-5415.2003.51467.x

[52] Leng S, Xue Q, Tian J, Huang Y, Yeh S, et al (2009) Associations of neutrophil and monocyte counts with frailty in community-dwelling disabled older women: results from the Women's Health and Aging Studies I. Experimental Gerontol 44: 511–516. 10.1016/j.exger.2009.05.005 19457449

[53] Collerton J, Martin-Ruiz C, Davies K, Hilkens C, Isaacs J, et al (2012) Frailty and the role of inflammation, immunosenescence and cellular ageing in the very old: cross-sectional findings from the Newcastle 85+ study. Mech Ageing Dev 133: 456–466. 22663935 10.1016/j.mad.2012.05.005

[54] Wang G, Kao W, Murakami P, Xue Q, Chiou R, et al (2010) Cytomegalovirus infection and the risk of mortality and frailty in older women: a prospective observational cohort study. Am J Epidemiol 171: 1144–1152. 20400465 10.1093/aje/kwq062 PMC2877470

[55] Mathei C, Vaes B, Wallemacq P, Degryse J (2011) Associations between cytomegalovirus infection and functional impairment and frailty in the BEFRAIL cohort. J Am Geriatr Soc 59: 2201–2208. 22092044 10.1111/j.1532-5415.2011.03719.x

[56] Berry S, Ngo L, Samelson E, Kiel D (2010) Competing risk of death: an important consideration in studies of older adults. J Am Geriatr Soc 58: 783–787. 20345862 10.1111/j.1532-5415.2010.02767.x PMC2873048

