# Supplementary figures and images for: Immune Biomarkers Predictive of Respiratory Viral Infection in Elderly Nursing Home Residents
Source: PLoS One. 2014 Oct 2;9(10):e108481. doi: 10.1371/journal.pone.0108481 (PMC4183538; doi:10.1371/journal.pone.0108481)

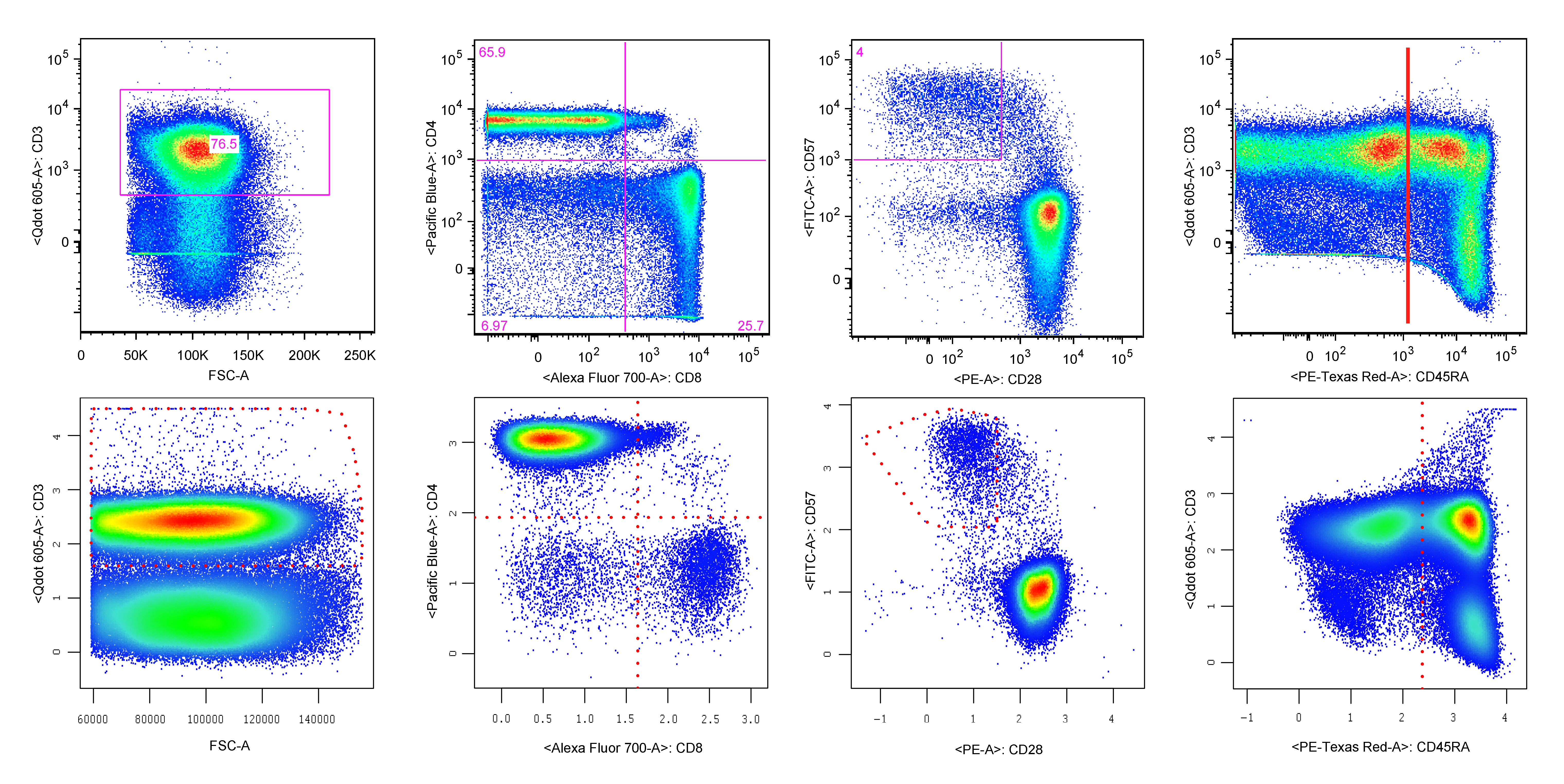

Supplement: Figure S1 — Comparison of automated versus manual gating for T-cell phenotypes. The same gating hierarchy was used for manual (top row) and automated (bottom row) approaches for the T-cell panel as described in the text. Similar results were obtained for both methods. (TIFF) [file pone.0108481.s001.tiff]
